# Autonomic and Vascular Control in Prehypertensive Subjects with a Family History of Arterial Hypertension

**DOI:** 10.5935/abc.20180006

**Published:** 2018-02

**Authors:** Josária Ferraz Amaral, Diana de Medeiros Andrade Borsato, Isabelle Magalhães Guedes Freitas, Edgar Toschi-Dias, Daniel Godoy Martinez, Mateus Camaroti Laterza

**Affiliations:** 1 Universidade Federal de Juiz de Fora (UFJF) Juiz de Fora, MG - Brazil; 2 Instituto do Coração (InCor) - Faculdade de Medicina da Universidade de São Paulo São Paulo, SP - Brazil

**Keywords:** Hypertension / genetic, Autonomic Nervous System, Risk Factors, Endothelium, Vascular / physiopathology

## Abstract

**Background:**

Individuals with a family history of systemic arterial hypertension (FHSAH) and / or prehypertension have a higher risk of developing this pathology.

**Objective:**

To evaluate the autonomic and vascular functions of prehypertensive patients with FHSAH.

**Methods:**

Twenty-five young volunteers with FHSAH, 14 normotensive and 11 prehypertensive subjects were submitted to vascular function evaluation by forearm vascular conductance(VC) during resting and reactive hyperemia (Hokanson®) and cardiac and peripheral autonomic modulation, quantified, respectively, by spectral analysis of heart rate (ECG) and systolic blood pressure (SBP) (FinometerPRO®). The transfer function analysis was used to measure the gain and response time of baroreflex. The statistical significance adopted was p ≤ 0.05.

**Results:**

Pre-hypertensive individuals, in relation to normotensive individuals, have higher VC both at rest (3.48 ± 1.26 vs. 2.67 ± 0.72 units, p = 0.05) and peak reactive hyperemia (25, 02 ± 8.18 vs. 18.66 ± 6.07 units, p = 0.04). The indices of cardiac autonomic modulation were similar between the groups. However, in the peripheral autonomic modulation, greater variability was observed in prehypertensive patients compared to normotensive individuals (9.4 [4.9-12.7] vs. 18.3 [14.8-26.7] mmHg^2^; p < 0.01) and higher spectral components of very low (6.9 [2.0-11.1] vs. 13.5 [10.7-22.4] mmHg^2^, p = 0.01) and low frequencies (1.7 [1.0-3.0] vs. 3.0 [2.0-4.0] mmHg^2^, p = 0.04) of SBP. Additionally, we observed a lower gain of baroreflex control in prehypertensive patients compared to normotensive patients (12.16 ± 4.18 vs. 18.23 ± 7.11 ms/mmHg, p = 0.03), but similar delay time (-1.55 ± 0.66 vs. -1.58 ± 0.72 s, p = 0.90).

**Conclusion:**

Prehypertensive patients with FHSAH have autonomic dysfunction and increased vascular conductance when compared to normotensive patients with the same risk factor.

## Introduction

Primary prevention has been recommended for individuals at increased risk for developing systemic arterial hypertension (SAH). Among them, individuals with a family history of SAH (FHSAH)^1,2^ and / or prehypertension^3^ stand out.

The reason for the increased susceptibility of hypertensive offspring to developing hypertension is not completely elucidated. However, studies indicate that autonomic abnormalities, such as increased sympathetic modulation,^4^ reduction of heart rate variability^4^ and reduction of baroreflex sensitivity^5^ are among the changes that may contribute to the onset of hypertension in normotensive children of hypertensive individuals. In addition, vascular abnormalities have also been considered as potential candidates for the onset of hypertension in this population.^6,7^

In prehypertensive patients, similar to those with FHSAH, dysfunctions^8,9^ and autonomic and vascular^10^ have also been pointed out as the main etiological factors of pressure elevation.

Although prehypertension has a strong genetic predisposition,^11,12^ the pathophysiological mechanisms responsible for pressure elevation in individuals with both risk factors, namely prehypertension and FHSAH, are not yet known. Therefore, this study aimed to evaluate the autonomic and vascular functions of prehypertensive individuals with FHSAH.

## Methods

### Sample

From the sample calculation performed based on the difference in sympathetic cardiac modulation of 0.31 ms^2^ between the means of the normotensive and prehypertensive groups,^13^ standard deviation of 0.21 ms^2^, alpha errors of 5% and beta of 20%, 7 individuals in each group would be needed. The sample consisted of 25 volunteers, subdivided according to blood pressure levels in the normotensive groups (SBP < 121 mmHg and / or DBP < 80 mmHg; n = 14) and prehypertensive (SBP between 121 and 139 mmHg and/or DBP between 80 and 89 mmHg, n = 11).^14^ All volunteers had FHSAH defined as father, mother, or both with a diagnosis of SAH, which was evaluated by means of a questionnaire.

Inclusion criteria adopted were age between 18 and 40 years, SBP lower than 140 mmHg, DBP lower than 90 mmHg and not involved in systematized physical exercises for at least six months prior to the research. In addition, only volunteers who had blood test results within 30 days prior to the start of the study in their medical records were included. Individuals with cardiometabolic diseases, smoking or drug treatment that could interfere with the cardiovascular system were not included.

This study was approved in the Committee of Ethics in Human Research of the HU / UFJF under the opinion nº 720/370. All volunteers signed the Free and Informed Consent Form.

### Measures and procedures

#### Anthropometry

For body mass and height measurements, we used, respectively, a scale with a precision of 0.1 kg and a stadiometer with a precision of 0.5 cm coupled to it (Líder®). The body mass index was calculated by dividing the body mass by the squared height (kg / m^2^).^15^ Waist circumference was measured using an inextensible metric tape (Cescorf®), with an accuracy of 0.1 cm. All of these variables were measured according to the criteria established by the American College of Sports Medicine.^16^

#### Blood pressure, heart rate and respiratory rate

With the volunteer at rest and in supine position, blood pressure (BP), heart rate and respiratory rate were monitored simultaneously for 15 minutes. Beat-to-beat BP was monitored by digital infrared photoplethysmography (FinometerPRO®) on the volunteer's dominant arm. Cardiac and respiratory rates were recorded continuously (Biopac®) using electrocardiogram in lead II and thoracic piezoelectric tape, respectively.

All acquired signals were reconstructed, digitized and recorded in a microcomputer with a sampling frequency of 1 kHz and 16-bit resolution for further analysis.

#### Forearm muscle blood flow and vascular conductance during rest and reactive hyperemia

Forearm muscle blood flow was evaluated using venous occlusion plethysmography (Hokanson® Plethysmograph). The volunteer was placed in dorsal decubitus position and the non-dominant forearm was raised above the level of the heart to ensure adequate venous drainage.

A silicon tube filled with mercury, connected to the low-pressure transducer and the plethysmograph, was placed around the volunteer's forearm, five centimeters away from the humeral-radial joint. One cuff was placed around the wrist and another at the top of the volunteer's arm. The wrist cuff was inflated at supra-systolic pressure level (200 mmHg) one minute before the measurements started and was kept inflated throughout the procedure. At 15-second intervals, the cuff placed on the arm was inflated at supra venous pressure (60 mmHg) for seven to eight seconds, then was deflated rapidly and maintained for the same period. This procedure totaled four cycles per minute.

The increase in tension in the silicone tube reflected the increase in forearm volume and, consequently, in an indirect way, increased forearm muscle blood flow, reported in ml/min/100 ml. The signal of the forearm muscle blood flow wave was acquired in real time in a computer through the *Non Invasive Vascular Program 3*.

The evaluation of peripheral vascular conductance was performed by dividing the peripheral vascular blood flow by the mean BP (mmHg), multiplied by 100 and expressed in "units".^17^

After measuring the forearm blood flow at rest for three minutes, the occlusion cuff positioned on the arm was inflated to 200 mmHg for five minutes. One minute before its deflation, the cuff placed on the wrist was also inflated to 200 mmHg remaining thus until the measurement was completed. After five minutes of occlusion, the arm cuff was rapidly deflated to induce reactive hyperemia and blood flow was recorded for the next three minutes, maintaining the cycle protocol, inflating to 60 mmHg for 10 seconds followed by 10 seconds of deflation.^18^ It was considered peak flow, the value of the first wave flow after the onset of reactive hyperemia.

During the evaluation of the blood flow of the forearm at rest and the protocol of reactive hyperemia, BP was measured beat-to-beat (FinometerPRO®). Additionally, during the rest period, cardiac output, left ventricular contractility (dP/dT maximum) and total peripheral resistance were also measured by the same equipment. In order to calculate the cardiac index, the cardiac output was corrected by the body surface area.^19^

#### Cardiac and peripheral autonomic modulation

The variabilities of the iRR, SBP and respiratory activity were evaluated in the frequency domain by autorregressive spectral analysis.

In stationary segments of 250 to 300 points, the time series of the iRR, respiration and SBP were decomposed into their frequency components by the autoregressive method using the Levinson-Durbin feature and the Akaike criterion for the choice of model order.^20^ This procedure allowed the automatic quantification of the central frequency and power of each relevant component of the spectrum. The spectral components of the frequency band between 0 and 0.04 Hz were considered very low frequency (VLF), the frequency band between 0.04 and 0.15 Hz was considered low frequency (LF) and the frequency band between 0.15 and 0.40 Hz, synchronized with respiration, considered high frequency (HF). Due to the short registration period, the VLF component of iRR variability does not present well-established physiological explanation.^21^ While the VLF of SBP variability seems to be related to myogenic vascular function.^22^ The LF component of iRR variability reflects, predominantly, cardiac sympathetic modulation and the HF component, synchronized with respiration, cardiac parasympathetic modulation.^21^ In the variability of SBP, the LF component quantifies the vasomotor sympathetic modulation, whereas the HF reflects the mechanical effect of respiration in the heart and vessels and does not represent an autonomic index.^23^

The spectral power of each component of the variability of iRR and SBP was calculated in absolute terms and in normalized units.^21^ The ratio between the LF and HF components of the iRR was calculated to quantify the cardiac sympathovagal balance.

#### Arterial baroreflex control

The gain and the time delay of response of the baroreflex control of the heart rate were measured by the analysis of the transfer function analysis using the bivariate autoregressive identification procedure.^24^ This procedure allowed the quantification of coherence, phase shift and gain among the time series of the iRR (output signal) and the SBP (input signal) as described by Freitas et al.^24^

In this study, the gain was calculated whenever the coherence between the signals was greater than 0.5 and the phase shift negative in the LF band, which indicates that the changes in the SBP preceded the changes in the iRR. In addition, it should be noted that the coherence, phase shift, gain and time delay of baroreflex control of heart rate were quantified at the central frequency corresponding to the maximum coherence within the LF band.

#### Experimental protocol

The evaluations were performed at the University Hospital of the Federal University of Juiz de Fora (HU-CAS), always in the morning. The volunteers were instructed not to ingest alcohol and / or caffeine and not to undertake vigorous physical activities within 24 hours prior to the evaluations as well as not eating fatty foods on the day of data collection.

The volunteers responded to the anamnesis that included the clinical data of the patients and their parents and were submitted to anthropometric evaluation. After the volunteers remained in the supine position for 10 minutes, simultaneous recording of heart rate, respiratory rate and BP was started for 15 minutes at rest. Then, the muscular blood flow of the forearm was measured during three minutes of rest and three minutes of reactive hyperemia.

### Statistical analysis

Data were presented as mean ± standard deviation of the mean or as median and interquartile range. To verify the normality of the distribution of all variables analyzed, the Shapiro-Wilk test was used. In addition, the assumption of homogeneity of variance was also verified by the Lèvene test. The distribution of the sexes between the groups was presented in absolute and percentage values. Fisher's exact test was used to verify the possible difference between the proportions of the sexes and of volunteers with both hypertensive parents in the groups.

The possible differences related to the demographic, clinical and autonomic characteristics of the groups were verified through the unpaired *Student t* test for the data that presented normal distribution and Mann-Whitney U for the variables that violated this assumption. Two-way analysis of variance for repeated measures was used to test for possible differences between groups in vascular conductance during resting and reactive hyperemia. The main and interaction effects were analyzed with Bonferroni confidence interval adjustment.

All statistical analyzes were performed using SPSS® software version 20. The statistical significance was p ≤ 0.05.

## Results

Of the 25 volunteers analyzed, one normotensive volunteer did not meet the acceptability criteria for the analysis of the cardiac and peripheral autonomic modulation, and one normotensive volunteer and two prehypertensive patients did not attend to the analysis of arterial baroreflex function.

**Figure 1:**
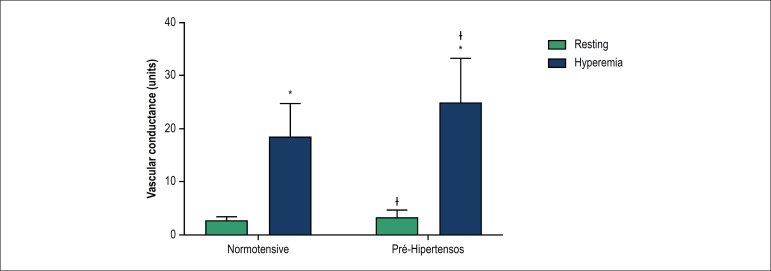
Vascular function. Data represented as mean ± standard deviation; ANOVA of two factors for repeated measures: *: significant differences in relation to rest; ᵻ: significant differences in relation to the normotensive group.

Table 1 shows the demographic and clinical characteristics of the groups evaluated. In addition to laboratory tests for glycemia, total cholesterol and triglycerides (Table 1), 13 normotensive volunteers and nine prehypertensive subjects measured serum creatinine levels (0.85 ± 0.21 and 0.94 ± 0.21 mg/dL, respectively), p = 0.350), and nine normotensive and seven prehypertensive patients measured serum uric acid levels (4.09 ± 1.55 and 4.84 ± 1.12 mg/dl, respectively, p = 296). No differences were observed between groups in any of the laboratory variables analyzed. Analysis of vascular function, measured by forearm vascular conductance during resting and reactive hyperemia, is shown in Figure 1. Vascular conductance increased during hyperemia in both the normotensive (p < 0.01) and prehypertensive (p < 0.01). In addition, although the prehypertensive group presented greater forearm vascular conductance both at rest (p = 0.05) and at the peak of reactive hyperemia (p = 0.04), this difference between groups tends to be more pronounced during the reactive hyperemia maneuver (interaction effect: p = 0.05).

**Table 1:** Demographic and clinical characteristics of the sample

Variable	Normotensive (n = 14)	Prehypertensive (n = 11)	p
Male gender n (%)	5 (35,7)	6 (54,5)	0,43^a^
Children of both hypertensive parents n (%)	4 (28,6)	5 (45,5)	0,43^a^
Age (years)	30 ± 6	29 ± 4	0,57^b^
BMI (kg/m^2^)	24 ± 4	25 ± 3	0,28^b^
Waist circumference (cm)	79 ± 11	82 ± 9	0,51^b^
Glycemia (mg/dl)	83 [80-93]	89 [83-93]	0,23^c^
Total Cholesterol (mg/dl)	177,9 ± 39,6	187,3 ± 29,7	0,53^b^
Triglycerides (mg/dl)	91,5 [57,9-131]	103,5 [63-148]	0,60^c^
SBP (mmHg)	116 [105-119]	128 [124-132]	< 0.01^c^
DBP (mmHg)	67 [60-71]	75 [71-75]	< 0.01^c^
Cardiac index (L/min/m^2^)	3,3 ± 0,3	3,7 ± 0,6	0,05^b^
Total peripheral resistance (mmHg/L)	15,0 [13,8-16,0]	13,8 [12,4-15,7]	0,15^c^
Cardiac contractility index (mmHg/s)	1113 ± 195	1340 ± 167	< 0,01^b^
Heart rate (bpm)	67 [ 63-69]	63 [ 62-76]	0,70^c^
Respiratory rate (ipm)	17 ± 2	17 ± 4	1,00^b^

Data presented as mean ± standard deviation of mean or median [interquartile range]; absolute value and percentage for males;

a Fisher's exact test;

b Unpaired Student t test;

c Mann-Whitney U-test; BMI: body mass index; SBP: systolic blood pressure; DBP: diastolic blood pressure.

**Figure 2:**
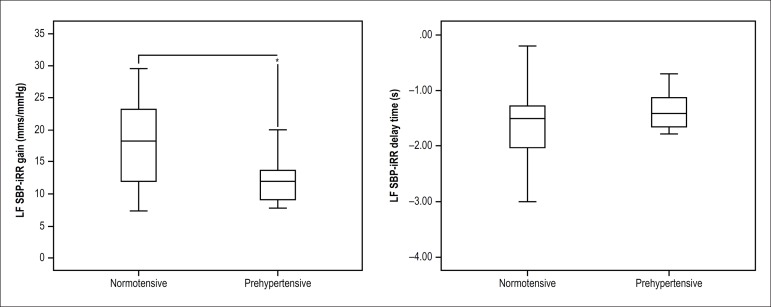
LF SBP-iRR gain and LF SBP-iRR delay time; Data represented in Box plot (minimum value, first quartile, median, third quartile and maximum value); iRR: RR interval; SBP: systolic blood pressure; LF: low frequency; Unpaired Student t test: *: significant difference in relation to the normotensive group (p = 0.03).

Indices of cardiac autonomic modulation were similar between the groups (Table 2). However, in the peripheral autonomic modulation, greater variability (VarianceSBP) and higher VLFSBP and LFSBP spectral components were observed in prehypertensive patients compared to normotensive patients (Table 2). Additionally, we observed a lower gain of baroreflex control in prehypertensive patients (LFSBP-iRR gain), but similar LFSBP-iRR delay time between groups (Figure 2).

**Table 2:** Cardiac and peripheral autonomic modulation

Variable	Normotensive (n = 13)	Prehypertensive (n = 11)	p
**Cardiac Modulation**			
Variance_IRR_ (ms^2^)	2050 [985-3264]	1718 [1067-3806]	0,50^b^
VLF_iRR_ (ms^2^)	905 ± 699	1178 ± 625	0,33^a^
LF_iRR_ (ms^2^)	565 [277-1067]	413 [263-1360]	0,98^b^
HF_iRR_ (ms^2^)	481 [212-897]	340 [195-606]	0,54^b^
LF_iRR_ (un)	51 ± 19	57 ± 17	0,46^a^
HFi_RR_ (un)	49 ± 19	43 ± 17	0,46^a^
LF/HF	0,90 [0,58-1,87]	1,52 [0,98-1,91]	0,50^b^
**Peripheral modulation**			
Variance _SBP_ (mmHg^2^)	9,4 [ 4,9-12,7]	18,3 [ 14,8-26,7]	< 0,01^b^
VLF_SBP_ (mmHg^2^)	6,9 [2,0-11,1]	13,5 [10,7-22,4]	0,01^b^
LF_SBP_ (mmHg^2^)	1,7 [1,0-3,0]	3,0 [2,0-4,0]	0,04^b^
HF_SBP_ (mmHg^2^)	2,0 [1,0-2,0]	1,0[1,0-2,5]	0,77^b^
Breathing			
LF (un)	0 [ 0-6]	0 [ 0-12]	0,92^b^
HF (un)	100 [94-100]	100 [88-100]	0,92^b^

Data presented as mean ± standard deviation of mean or median [interquartile range];

a unpaired Student t test;

b Mann-Whitney U-test; iRR: RR interval; SBP: systolic blood pressure; VLF : very low frequency; LF: low frequency; HF: high frequency; un: standard units.

Table 3 shows the central frequency, phase shift and coherence of the LF component of the SBP-iRR relationship, as well as the central frequency and coherence of the LF and HF components of the relationship between respiratory activity and the iRR.

**Table 3:** Arterial baroreflex function

Variable	Normotensive (n = 13)	Prehypertensive (n = 9)	p
**SBP-iRR**			
LF Central frequency (Hz)	0,10 ± 0,02	0,10 ± 0,01	0,58^a^
LF Phase shift (rad)	-0,96 ± 0,33	- 0,94 ± 0,31	0,90^a^
LF Coherence	0,85 ± 0,08	0,79 ±0,14	0,15^a^
**Resp-iRR**			
LF Central frequency (Hz)	0,14 [0,10-0,15]	0,10 [0,07-0,12]	0,08^b^
LF Coherence	0,47 ± 0,19	0,42 ± 0,16	0,56^a^
Central frequency HF (Hz)	0,29 [0,28-0,30]	0,32 [0,27-0,33]	0,42^b^
HF Coherence	0,96 [0,91-0,98]	0,93 [0,92-0,95]	0,22^b^

Data presented as mean ± standard deviation of mean or median [interquartile range];

a unpaired Student t test;

b Mann-Whitney U-test; iRR-RR interval; SBP: systolic blood pressure; LF: low frequency; HF: high frequency.

## Discussion

The main finding of this study is that peripheral autonomic dysfunction precedes the possible vascular dysfunction in prehypertensive individuals with FHSAH.

As expected, the prehypertensive group had higher SBP and DBP. Since blood pressure values are determined by cardiac output and peripheral vascular resistance, in this study, increased cardiac output by increasing systolic volume, possibly modulated by increased cardiac contractility, appears to be related to blood pressure elevation, since both heart rate and peripheral vascular resistance were similar between the groups. Similar results were obtained by Davis et al.,^12^ who also observed elevation of cardiac index and cardiac contractility, but similar peripheral resistance, in young prehypertensive individuals when compared to normotensive ones. Thus, although the typical hemodynamic finding of hypertension is elevation of peripheral resistance, elevation of cardiac output appears to be responsible for pressure elevation in the early stages of disease development.^25^

In addition, studies have shown impairment in the vascular function of prehypertensive patients such as reduction of endothelium-dependent vasodilation, assessed by the infusion of acetylcholine,^10^ reduction of the plasma concentration of vasodilatory substances, such as nitric oxide^26^ and elevation of vasoconstrictors such as endothelin-1.^10,26^ However, in this study, we observed greater forearm vascular conductance in both the rest and the peak of reactive hyperemia in the prehypertensive patients when compared to the normotensive ones. Other studies, also using the venous occlusion plethysmography technique, obtained controversial results regarding the vascular function of prehypertensive patients. For example, Schwartz et al.^27^ evaluated the resting forearm vascular conductance of normotensive and prehypertensive young men and did not observe differences between groups. Beck et al.^28^ evaluated youngsters of both sexes and observed lower vascular conductance in prehypertensive patients in relation to the normotensive ones.

Already during the maneuver of reactive hyperemia, Beck et al.^26^ and Beck et al.,^28^ in contrast to the results of this study, observed a lower peak flow in prehypertensive patients using, respectively, venous occlusion plethysmography and high-resolution ultrasound. The differences between the results of this study and the others may be related to the characteristics of the population studied, such as the presence of FHSAH in both groups, since individuals with this risk factor have demonstrated vascular dysfunction in several studies.^6,7^ In addition to FHSAH, pre-hypertensive volunteers in this study had higher cardiac and contractility rates, which may have triggered a local vasodilatory homeostatic response in an attempt to alleviate pressure elevation,^12^ although this mechanism failed systematically in view of the fact that no difference was observed between groups in peripheral vascular resistance. No studies were found that investigated the association between cardiac and contractility indices and vascular conductance in prehypertensive patients. In hypertensive patients with hyperkinetic circulation, characterized by elevation of cardiac index and mean arterial pressure, Stevo et al.^29^ observed greater forearm muscle blood flow compared to normotensive individuals. However, in this study the calculation of vascular conductance was not performed. Thus, future studies should investigate the association between these variables in pre-hypertensive individuals with a family history of arterial hypertension.

According to Davis et al.,^12^ BP elevation in prehypertension results from hereditary disorders that present a set of genetic determinants and pathogenic traits that act on hemodynamic and autonomic events in series and trigger the SAH. In this scenario, autonomic alterations appear to be the first changes observed in prehypertensive patients.^12^ However, although changes in the spectral indices of cardiac autonomic modulation in prehypertensive patients have been demonstrated in other studies,^8,30^ in this one, they were not observed. Lin et al.,^13^ who also observed LF and HF components in normalized units, as well as the LF/HF ratio of heart rate variability, similar among normotensive and prehypertensive youngsters, reported results similar to ours. A possible explanation for these contradictory results is the population studied. In this study, we evaluated normotensive and pre-hypertensive individuals with FHSAH, while the other studies did not control the distribution of this risk factor in the analyzed groups. Thus, since alterations in cardiac autonomic modulation have been demonstrated in normotensive individuals with hypertensive father and / or mother,^4,5^ further studies are needed to elucidate these alterations in individuals who have both risk factors, prehypertension and FHSAH.

Regarding the autonomic peripheral modulation, in this study we verified dysfunctions in this system in the prehypertensive individuals. We observed a higher LF component of SBP variability in prehypertensive patients compared to normotensive patients, which shows a greater performance of vascular tone sympathetic modulation as well as myogenic vascular function in this population.^23^ Similar results were reported by Hering et al.^31^ and Seravalle et al.^9^ who evaluated individuals with normal-high pressure and also observed greater peripheral sympathetic modulation, assessed by the microneurography technique, in these individuals when compared to normotensive individuals.

The variability of SBP, as well as elevation of pressure levels, has been recognized as an important risk factor for target organ damage.^32^ In this study, pre-hypertensive individuals presented greater variance of SBP in relation to normotensive individuals, corroborating the results of Duprez et al.^33^ However, these authors did not report the FHSAH of study participants.

BP fluctuations are triggered by multiple systems including the renin-angiotensin system, baroreflex, myogenic vascular response, and release of nitric oxide.^23^ Thus, the elevations of the LF and VLF components observed in this study may be related to the increase in SBP variability through changes in myogenic vascular function.^23^ The HF component, which appears to be related to endothelial nitric oxide^23^, was similar between the groups and did not appear to be involved in increased pressure variability.

In addition, this study demonstrated a reduction in the baroreflex control of heart rate in prehypertensive individuals when compared to normotensive individuals, a factor that may also be related to the increased pressure variability and peripheral sympathetic modulation observed.^34^ The results of this study corroborate the findings of previous studies^9,11,13^ that also observed reduction of baroreflex sensitivity in prehypertensive patients. However, this is the first to demonstrate autonomic changes in prehypertensive patients with FHSAH in relation to normotensive individuals with the same risk factor.

In addition to sensitivity, the response time of the baroreflex can also determine the efficiency of this reflex.^35^ In this study, we verified the baroreflex response time preserved in prehypertensive patients. This characteristic of the baroreflex is mainly affected by changes in cardiac parasympathetic nervous modulation,^36^ a change that was not observed in the prehypertensive patients evaluated in this study. Therefore, it is possible that the response time of the baroreflex could be affected later in the course of pressure rise and development of hypertension and that in the prehypertension phase only the reduction of the gain contributes to the reduction of the efficiency of this reflex. In addition, the fact that the volunteers in this study have FHSAH may be related to the observed results. No studies were found to investigate this time delay of the baroreflex effector response in prehypertensive individuals, as well as in children with hypertensive parents, which made difficult to compare our results.

This study demonstrated that prehypertensive youngsters with FHSAH present autonomic dysfunction and vascular function similar to normotensive with the same risk factor. Thus, the results of this study emphasize the importance of preventive intervention with measures aimed at attenuating this dysfunction and, consequently, acting on the prevention of hypertension in this population. In this sense, physical exercise has been considered effective since it acts in a beneficial way in multiple physiological systems.^37^ In addition, the benefits of regular aerobic physical exercise in the attenuation of autonomic dysfunction have already been demonstrated both in prehypertensive patients^37^ and in the descendants of hypertensive parents,^38^ which leads us to believe that individuals with both risk factors may also benefit from the effects of this practice.

### Limitations

The diagnosis of SAH of the parents of the volunteers of this study was self-reported. Although self-report has been used in many studies,^38,39^ future research should include detailed medical evaluation of the parents. The presence of renal diseases was not an exclusion criterion in this study, since all the necessary tests to exclude safely this characteristic were not performed. In spite of this, all the volunteers declared that they did not have a diagnosis of renal diseases and those who did the creatinine and uric acid tests presented normal values for these variables. Additionally, the women in this study were not evaluated during the same period of the menstrual cycle, a fact that may also be a limitation of this work. However, Jarvis et al.^40^ and Carter et al.^41^ observed no influence of the ovarian cycle phase on sympathetic modulation, heart rate and BP during rest in young women. Despite the limitations pointed out, the great strength of this study is the fact that we evaluated young adults, without medication and with similar glycemic and lipid profile.

## Conclusion

We conclude that prehypertensive patients with FHSAH have autonomic dysfunction, characterized by increased peripheral sympathetic modulation and reduced baroreflex control of heart rate, and increased vascular conductance when compared to normotensive patients with the same risk factor.

## References

[1] Mitsumata K, Saitoh S, Ohnishi H, Akasaka H, Miura T (2012). Effects of parental hypertension on longitudinal trends in blood
pressure and plasma metabolic profile mixed-effects model
analysis. Hypertension.

[2] Wang NY, Young JH, Meoni LA, Ford DE, Erlinger TP, Klag MJ (2008). Blood pressure change and risk of hypertension associated with
parental hypertension: the Johns Hopkins Precursors Study. Arch Intern Med.

[3] Collier SR, Landram MJ (2012). Treatment of prehypertension: lifestyle and/or
medication. Vasc Health Risk Manag.

[4] Francica JV, Heeren MV, Tubaldini M, Sartori M, Mostarda C, Araujo RC (2013). Impairment on cardiovascular and autonomic adjustments to maximal
isometric exercise tests in offspring of hypertensive
parents. Eur J Prev Cardiol.

[5] Lénárd Z, Studinger P, Mersich B, Pavlik G, Kollai M (2005). Cardiovagal autonomic function in sedentary and trained offspring
of hypertensive parents. J Physiol.

[6] Boutcher YN, Park YJ, Boutcher SH (2009). Vascular and baroreceptor abnormalities in young males with a
family history of hypertension. Eur J Appl Physiol.

[7] Evrengul H, Tanriverdi H, Kilic ID, Dursunoglu D, Ozcan EE, Kaftan A (2012). Aortic stiffness and flow-mediated dilatation in normotensive
offspring of parents with hypertension. Cardiol Young.

[8] Pal GK, Adithan C, Amudharaj D, Dutta TK, Pal P, Nandan PG (2011). Assessment of sympathovagal imbalance by spectral analysis of
heart rate variability in prehypertensive and hypertensive patients in
Indian population. Clin Exp Hypertens.

[9] Seravalle G, Lonati L, Buzzi S, Cairo M, Quarti Trevano F, Dell'Oro R (2015). Sympathetic nerve traffic and baroreflex function in optimal,
normal, and high-normal blood pressure states. J Hypertens.

[10] Weil BR, Westby CM, Greiner JJ, Stauffer BL, DeSouza CA (2012). Elevated endothelin-1 vasoconstrictor tone in prehypertensive
adults. Can J Cardiol.

[11] Pal GK, Adithan C, Umamaheswaran G, Pal P, Nanda N, Indumathy J (2016). Endothelial nitric oxide synthase gene polymorphisms are
associated with cardiovascular risks in prehypertensives. J Am Soc Hypertens.

[12] Davis JT, Rao F, Naqshbandi D, Fung MM, Zhang K, Schork AJ (2012). Autonomic and hemodynamic origins of pre-hypertension: central
role of heredity. J Am Coll Cardiol.

[13] Lin G, Xiang Q, Fu X, Wang S, Wang S, Chen S (2012). Heart rate variability biofeedback decreases blood pressure in
prehypertensive subjects by improving autonomic function and
baroreflex. J Altern Complement Med.

[14] Malachias MV, Souza WK, Plavnik FL, Rodrigues CI, Brandão AA, Neves MF, Sociedade Brasileira de Cardiologia (2016). 7a Diretriz Brasileira de hipertensão
arterial. Arq Bras Cardiol.

[15] Quetelet A (1870). Anthropométrie ou mesure des différentes facultés
de l'homme.

[16] American College of Sports Medicine (2007). Diretrizes do ACSM para os testes de esforço e sua
prescrição.

[17] Tarvainen MP, Ranta-Aho PO, Karjalainen PA (2002). An advanced detrending method with application to HRV
analysis. IEEE Trans Biomed Eng.

[18] Bousquet-Santos K, Soares PP, Nobrega AC (2005). Subacute effects of a maximal exercise bout on
endothelium-mediated vasodilation in healthy subjects. Braz J Med Biol Res.

[19] DuBois D, DuBois EF (1989). A formula to estimate the approximate surface area if height and
weight be known. Nutrition.

[20] Pagani M, Lombardi F, Guzzetti S, Rimoldi O, Furlan R, Pizzinelli P (1986). Power spectral analysis of heart rate and arterial pressure
variabilities as a marker of sympatho-vagal interaction in man and conscious
dog. Circ Res.

[21] Task Force of the European Society of Cardiology, North American Society of Pacing and Electrophysiology (1996). Heart rate variability: standards of measurement, physiological
interpretation and clinical use. Circulation.

[22] Hocht C (2013). Blood pressure variability: prognostic value and ¨ therapeutic
implications. ISRN Hypertension.

[23] Stauss HM (2007). Identification of blood pressure control mechanisms by power
spectral analysis. Clin Exp Pharmacol Physiol.

[24] Freitas IM, de Almeida LB, Pereira NP, de Carvalho Mira PA, de Paula RB, Martinez DG (2017). Baroreflex gain and vasomotor sympathetic modulation in resistant
hypertension. Clin Auton Res.

[25] Post WS, Larson MG, Levy D (1994). Hemodynamic predictors of incident hypertension. The Framingham
Heart Study. Hypertension.

[26] Beck DT, Casey DP, Martin JS, Emerson BD, Braith RW (2013). Exercise training improves endothelial function in young
prehypertensives. Exp Biol Med.

[27] Schwartz CE, Durocher JJ, Carter JR (2011). Neurovascular responses to mental stress in prehypertensive
humans. J Appl Physiol (1985).

[28] Beck DT, Martin JS, Casey DP, Braith RW (2014). Exercise training improves endothelial function in resistance
arteries of young prehypertensives. J Hum Hypertens.

[29] Julius S, Krause L, Schork NJ, Mejia AD, Jones KA, van de Ven C (1991). Hyperkinetic borderline hypertension in Tecumseh,
Michigan. J Hypertens.

[30] Wu JS, Lu FH, Yang YC, Lin TS, Chen JJ, Wu CH (2008). Epidemiological study on the effect of pre-hypertension and
family history of hypertension on cardiac autonomic function. J Am Coll Cardiol.

[31] Hering D, Kara T, Kucharska W, Somers VK, Narkiewicz K (2013). High-normal blood pressure is associated with increased resting
sympathetic activity but normal responses to stress tests. Blood press.

[32] Kouchaki Z, Butlin M, Qasem A, Avolio AP (2016). Quantification of peripheral and central blood pressure variability
using a time-frequency method.

[33] Duprez DA, De Sutter JH, De Buyzere ML, Rietzschel ER, Rimbaut S, Kaufman JM (1995). Renin-angiotensin-aldosterone system, RR interval, and blood
pressure variability during postural changes in borderline arterial
hypertension. Am J Hypertens.

[34] Wei X, Fang X, Ren L, Meng Y, Zhang Z, Wang Y (2013). The effect of baroreflex function on blood pressure
variability. Int J Clin Med.

[35] Cevese A, Gulli G, Polati E, Gottin L, Grasso R (2001). Baroreflex and oscillation of heart period at 0.1 Hz studied by
a-blockade and cross-spectral analysis in healthy humans. J Physiol.

[36] Keyl C, Schneider A, Dambacher M, Bernardi L (2001). Time delay of vagally mediated cardiac baroreflex response varies
with autonomic cardiovascular control. J Appl Physiol.

[37] Collier SR, Kanaley JA, Carhart Jr R, Frechette V, Tobin MM, Bennett N (2009). Cardiac autonomic function and baroreflex changes following 4
weeks of resistance versus aerobic training in individuals with
pre-hypertension. Acta Physiol (Oxf).

[38] Goldberg M, Boutcher S, Boutcher Y (2012). The effect of 4 weeks of aerobic exercise on vascular and
baroreflex function of young men with a family history of
hypertension. J Hum Hypertens.

[39] Boutcher YN, Hopp JP, Boutcher SH (2011). Acute effect of a single bout of aerobic exercise on vascular and
baroreflex function of young males with a family history of
hypertension. J Hum Hypertens.

[40] Jarvis SS, VanGundy TB, Galbreath MM, Shibata S, Okazaki K, Reelick MF (2011). Sex differences in the modulation of vasomotor sympathetic
outflow during static handgrip exercise in healthy young
humans. Am J Physiol Regul Integr Comp Physiol.

[41] Carter JR, Lawrence JE (2007). Effects of the menstrual cycle on sympathetic neural responses to
mental stress in humans. J Physiol.

